# Enhanced larval supply and recruitment can replenish reef corals on degraded reefs

**DOI:** 10.1038/s41598-017-14546-y

**Published:** 2017-10-25

**Authors:** Dexter W. dela Cruz, Peter L. Harrison

**Affiliations:** 1 Marine Ecology Research Centre, IDEAS Research Institute, School of Environment, Science and Engineering, Southern Cross University, Lismore, New South Wales 2480 Australia; 2 The Marine Science Institute, College of Science, University of the Philippines, Diliman, Quezon City 1101 Philippines

## Abstract

Reef-building corals have essential roles in reef ecosystems but are highly susceptible to disturbances. Increasing anthropogenic disturbances are eroding coral community resilience, leading to declining reef ecosystem function and status globally. Successful reproduction and recruitment are essential for restoring coral populations but recruitment-limitation can constrain recovery. We supplied ~400,000 *Acropora tenuis* larvae in fine-mesh enclosures on each of four larval-enhancement plots, comprising natural reef substrata and ten settlement tiles, on degraded reef areas in the northwestern Philippines. Initial mean total settlement on tiles in larval-enhancement plots was high (255.3 ± 68.6), whereas no larvae settled in natural control plots. Recruit survivorship began stabilising after five months, with juveniles becoming visible by eye at nine months. After three years a mean of 2.3 m^−2^ colonies survived within each larval-enhancement plot. Most colonies grew rapidly (16.1 ± 0.7 cm mean diameter) and spawned successfully at three years, thereby quickly re-establishing a breeding population. In contrast, natural recruitment failed to produce any new visible *A*. *tenuis* colonies. These results demonstrate that mass larval settlement can rapidly enhance recruitment and coral recovery on degraded reef areas, and provides an important option for active reef restoration where larval supply and recruitment success are limiting.

## Introduction

Coral reefs are facing intensifying anthropogenic impacts^1–3^ resulting in reef-building coral populations and live coral cover declining in many reef regions^4–6^. More than 60 percent of the world’s coral reefs are degraded or seriously threatened with loss in coming decades^2,7^. Human threats to coral reefs are particularly severe in the Coral Triangle region that encompasses the global center of marine biodiversity^8^. It is estimated that nearly 95 percent of coral reefs in the Southeast Asia region are threatened by human activities and about 50 percent of these reefs are in the high or very high threat categories, with Indonesia and the Philippines having the largest areas of threatened reefs^7^. Reef degradation directly threatens the extraordinary biodiversity and immense ecological and socio-economic values of reefs that support many millions of people^7,9^. Although it is not currently feasible to restore all degraded reefs, mitigating the causes of degradation and initiating restoration of corals on reefs that can be managed are becoming increasingly urgent.

Successful reproduction and recruitment are critical for the maintenance and recovery of scleractinian coral species and underpin reef resilience^10,11^. However, ongoing declines in coral abundance and reproductive biomass can lead to loss of breeding colonies and decreasing reproductive success (critical depensation) resulting in recruitment limitation or failure^12–14^.

Active coral restoration can enhance coral cover and abundance, but current restoration methods have not yet been effective at larger scales needed to halt or reverse the decline in reef coral communities^15,16^. Most coral restoration studies have used smaller-scale asexual fragmentation of adult colonies in combination with various transplantation and nursery techniques, but these are relatively labour-intensive and expensive^17,18^. Fragmentation damages donor colonies and increases disease risk, and most coral fragmentation studies have used fragments with limited genetic diversity which constrains their resistance to future stress disturbances^16,18,19^. Alternatively, mass larval enhancement using millions of sexually-derived larvae resulting from large-scale spawning events of highly fecund corals^20,21^ has strong potential for scaling-up restoration efforts to larger reefal scales^10^. Mass larval enhancement also has significant advantages of providing greater genotypic diversity among recruits that is likely to improve adaptive and evolutionary potential^22^ and increase resistance to future disturbances^23^, thereby strengthening reef community resilience and recovery rates.

To date, results from only two small-scale *in situ* field trials using mass coral larval settlement have been published, and both studies used reef areas adjacent to healthy coral communities in Marine Protected Areas^24,25^. Heyward *et al*.^24^ used *Acropora* larvae sampled from coral spawn slicks and reared in small 1.8 m floating culture ponds to ‘seed’ small 1.8 × 1 m reef areas at Ningaloo Reef, Western Australia^24^. After four weeks more than 6,500 acroporid coral recruits were growing on conditioned terracotta tiles in the larval ‘seeded’ areas, whereas settlement on control tiles and natural recruitment rates were up to 100-fold lower^24^. However, subsequent survival or growth of the recruits was not monitored. Hence, longer-term recruitment outcomes from this study were not determined. In Palau, ~1 million *ex situ* cultured *Acropora digitifera* larvae were used to ‘seed’ replicate concrete pallet balls for 24 hours resulting in significantly higher acroporid recruitment on fibre-cement tiles attached to ‘seeded’ pallet balls compared with unseeded control tiles after 5 weeks^25^. Monitoring after 30 weeks and ~13 months showed no significant differences in acroporid recruit densities between ‘seeded’ and control treatments due to high post-settlement mortality of *A*. *digitifera* recruits and high rates of natural acroporid coral recruitment in this healthy reef system^25^.

Here, we evaluate for the first time the longer-term effects of mass coral larval settlement directly on degraded reef areas in the northwestern Philippines where coral cover is low and natural recruitment is severely limited. Specifically, we demonstrate the effectiveness of using mass larval enhancement to initiate coral recovery by monitoring settlement, survival, growth and onset of sexual reproduction of *Acropora tenuis* recruits on coral recruitment tiles and natural reef substrata for three years.

## Methods

### Experimental Design

This experiment was designed to test the effect of supplying large numbers of coral larvae on replicate degraded reef areas during a five-day larval settlement period to quantify initial larval settlement and longer-term recruitment outcomes. Long-term monitoring over three years enabled subsequent patterns of post-settlement survival, growth and onset of sexual reproduction of coral recruits in larval enhanced plots versus control plots without larval provision to be compared.

### Site selection and reef benthic community status

**Figure 1:**
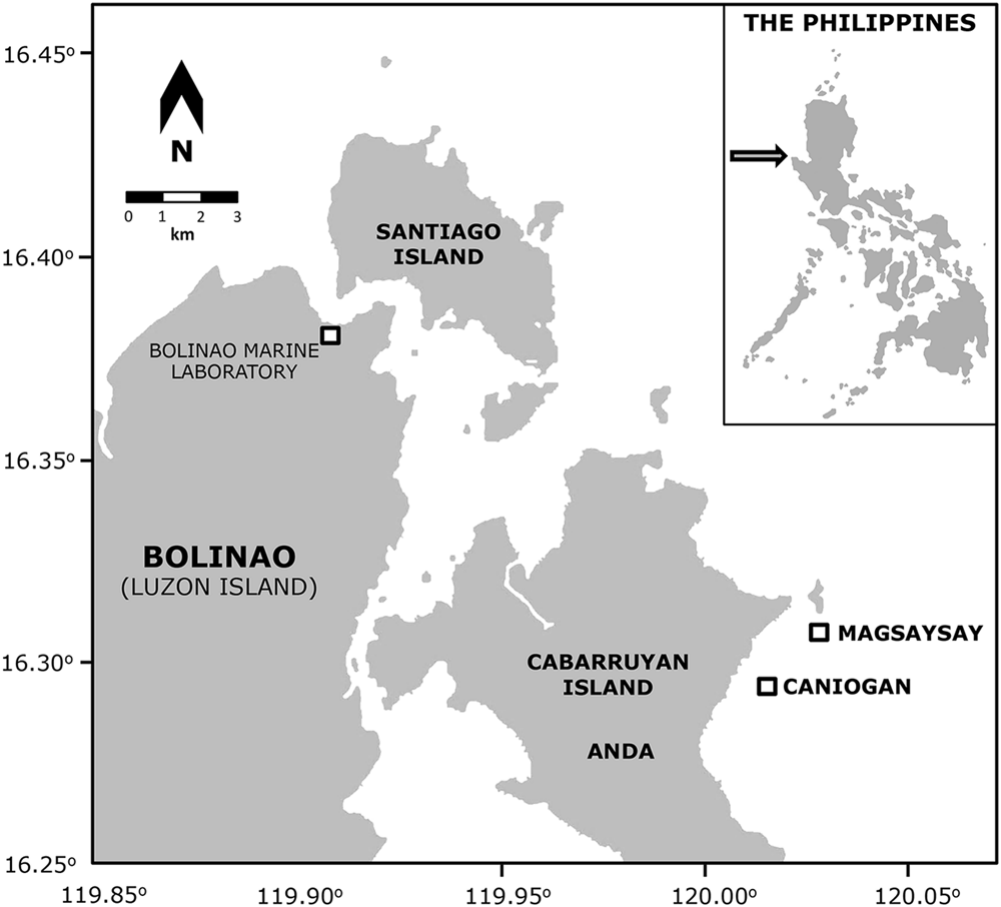
Locations of the experimental larval-enhanced and control plots (Magsaysay Reef) and the source of *A*. *tenuis* colonies (Caniogan Reef) in the Bolinao-Anda Reef Complex, northwestern Philippines. (Figure created with Adobe Illustrator CS6 http://www.adobe.com/uk/products/illustrator.html).

Prior to the larval enhancement experiment, degraded reef areas at 3–4 m depth were identified at the Magsaysay reef in the Lingayen Gulf, Anda, Pangasinan, Northern Luzon, Philippines (16°19’36” N, 120°02’01” E; Fig. 1). Relatively high 30–50% coral cover was recorded in surveys on reefs in the Bolinao-Anda Reef Complex (BARC), Pangasinan, up to 1981^26^. However, reef status and water quality in the Lingayen Gulf have been declining since the early 1980s, coinciding with increased fishing pressure including destructive blast fishing, and aquaculture development^27^. Live coral cover on reefs in Bolinao declined from about 41% mean cover in the late 1908s to less than 22% mean cover by 1999^27^. The coral community^28^ in the Magsaysay reef area was subsequently severely impacted by a Crown-of-thorns starfish outbreak in 2007 (DWdC, pers. obs.) and previous extensive blast fishing which no longer occurs^27^. For the present study, a total of eight 6 × 4 m reef plots (about 2–4 m apart) were haphazardly selected on the reef and demarcated using steel bars. Four randomly selected plots were treated with larvae (larval-enhanced) and the other four plots were controls – without provision of larvae. Prior to the larval enhancement experiment, photographs of the benthic communities within each plot were taken using a 1 × 1 m frame and analysed using CPCe^29^ to quantify benthic cover of corals and other benthos and to determine the reef community status in the area. A total of 10 random points were generated and the benthic category underlying each point was scored in each of the 24 frames taken in each of the plots. There were no *A*. *tenuis* adult colonies or visible recruits from previous spawning events present inside the plots prior to the larval enhancement activity.

### Settlement tiles

Ten biologically conditioned 10 × 10 cm natural coral settlement and recruitment tiles cut from dead table *Acropora* with varying thickness (about 3–4 cm average) were deployed inside each of the eight plots just prior to the larval enhancement experiment to quantify the initial settlement rates of the *A*. *tenuis* larvae. Each tile was identified with a coded tag, and was attached onto a stainless steel post projecting from a base plate attached to the reef, with a 0.5 cm gap above the base plate to enable larval settlement on all surfaces^30^. Dead table *Acropora* pieces used to produce the tiles were collected from the intertidal zone beside Cory reef rubble bar near the experimental site (Fig. 1). The tiles were conditioned for a month in aquaculture tanks with flow-through seawater and aeration at the Bolinao Marine Laboratory (BML) of The Marine Science Institute (University of the Philippines) prior to use on the reef plots, and were examined to ensure no coral recruits were present prior to their use.

To estimate the total surface area of the irregular surfaces of the tiles, 30 representative tiles were 3D scanned using a David^®^ SLS-1 structured white light technology scanning system. The scanner had a mesh density up to 1,200,000 vertices per scan with resolution precision up to 0.1% of scan size down to 0.06 mm. Each tile was scanned and shot in all angles using a 360° turntable and each shot was fused to form a digitised 3D object. The formed 3D object was then converted to a stereolithography (STL) file that was further refined using 3D modelling software (viz., Zbruzh^™^ and Blender^™^). The surface area of the 3D image of the tiles was analysed and calculated using netFabb^®^ software. As there was minimal variation in the surface area of the scanned tiles (mean: 360 ± 3.7 SE cm^2^), larval settlement and recruitment was expressed as numbers of recruits per tile.

### Coral collection and larval culture

Thirty gravid colonies of *Acropora tenuis* (minimum diameter of 15–20 cm) were collected from the reef ~2–4 m deep in Caniogan, Anda, Pangasinan (Fig. 1; 16°19’36” N, 120°02’01” E) a week before the full moon of April 2013. This species was selected for this study because it is relatively common in the Bolinao-Anda Reef Complex in the northwestern Philippines, and larvae from this species have been successfully cultured in previous experiments^31–33^. Furthermore, *Acropora tenuis* was chosen for this study as it has a well-defined and characteristic morphology that enables colonies to be readily identified and distinguished from other species underwater^34^ thereby enabling early detection, identification and monitoring of recruits resulting from larval settlement. Prior to collection, colonies were sampled by carefully breaking a few branches to determine the presence of mature oocytes, as indicated by their pink colouration^35^. Gravid colonies were carefully transported in seawater to the BML aquaculture facility for *ex situ* spawning and gamete collection.

Colonies were maintained in a healthy condition in concrete tanks with flow-through seawater and aeration and were monitored periodically at night to check for setting and spawning behaviours^20,36,37^. Gametes from major spawning events involving 30 colonies that spawned between 1830 and 1900 h on April 29 and 30 2013 (3 and 4 nights after full moon, respectively) were collected for use in this experiment. Gamete collection and larval culture followed standard methods with minor modifications^16,38–40^.

Many thousands of spawned egg-sperm bundles were skimmed off the water surface and transferred to a fertilization container in 10 L of 1 µm filtered seawater. Gamete bundles were gently agitated to facilitate egg and sperm separation and to maximize subsequent cross-fertilization^41^. After 1 hour, seawater containing excess sperm was siphoned off (sperm-washing) beneath the floating eggs and new filtered seawater was slowly added. This process was repeated three times to remove excess sperm that may degrade water quality during larval culture and to prevent polyspermy^41^. Subsamples of embryos and eggs were collected and examined after a further hour under a stereomicroscope to determine percentage fertilization.

Developing embryos were transferred into 13 large rearing tanks (each containing >1,000 L of seawater) at water surface densities between 4–5 embryos cm^−2^ for 24 h. To maintain embryos and larvae in a healthy condition, aeration was supplied 24 h after fertilization and 50 L of new filtered seawater was added daily to tanks with developing larvae. Spawned coral colonies were returned in a healthy condition and reattached onto the reef using underwater adhesive (a mixture of cement and epoxy).

At 4 d post-fertilization, competent actively swimming larvae were collected using a plankton net sieve (60 µm mesh size) and transferred to a holding basin. The total number of larvae was estimated using three replicate 60 ml sub-samples taken from the basin after the larvae had been concentrated and thoroughly mixed throughout the water column to distribute them evenly, and then the larvae were distributed equally into twelve strong 40 × 50 cm 20 L plastic bags. Oxygen was supplied into each bag before it was sealed for transport to the field for the larval enhancement experiment at Magsaysay reef.

### Coral larval enhancement

Organza-hapa mesh enclosures were used to retain the larvae inside the four larval enhancement plots during the 5-day larval settlement period. The organza cloth (100–150 µm mesh opening) was sewn to form 6 × 4 m enclosures, with hapa nylon net (500 µm mesh) as a second, outer layer for additional support. This mesh enclosure assembly can effectively retain *A*. *tenuis* larvae whose diameters are 300–500 µm^42^. Cylindrical 20 g weights (1.75 × 4 cm) were inserted along the lower edge of the mesh to firmly hold the mesh enclosure in place on the reef and prevent larvae from escaping during the settlement period. The mesh enclosures covered the top and sides of each site and extended up to 75 cm from the reef with the bottom open to the reef to enable larvae to settle, and the corners were secured using steel reinforcing bars driven into the substratum. An estimated 410,670 ± 26,354 larvae were introduced into the mesh enclosure on each of the four larval-enhanced plots through re-sealable openings distributed over the upper areas of the mesh enclosures. Control plots were also covered by organza-hapa mesh enclosures during this period but no larvae were introduced. All living corals inside the plots (between 10 – 30 colonies up to 80 cm diameter per plot) were carefully covered with thin plastic mesh with 1 cm mesh openings to avoid the mesh enclosures from becoming caught and ripping and damaging the coral tissues.

Five days after the provision of larvae inside the mesh enclosures, the enclosures and the protective plastic mesh around living corals were removed from all plots. All settlement tiles were then carefully collected and kept submerged in clean seawater while carefully taken to the BML laboratory to monitor the initial numbers of settled coral spat on each tile under a stereoscope while the tiles remained submerged in seawater. Each coral recruit was recorded and carefully mapped to facilitate repeated monitoring at 2, 5, 9, 12, 15, 17, 19, 21, 23, 31, 34 and 35 months after settlement. The tiles were returned immediately to their specific location and correct orientation within each experimental plot after each monitoring period. Where required, small clumps of algae were periodically removed from the tiles to avoid recruits being overgrown by algae.

### Coral recruits on reef substrata

The process of larval settlement includes the initial attachment of competent larvae onto substrata and their metamorphosis into a coral polyp (spat), while recruitment refers to the subsequent life stage of the coral following post-settlement survival and growth to become new members of the population^21,43^. Corals are initially small and invisible cryptic recruits and subsequently grow to become larger visible colonies that are often referred to as juveniles^44^. *Acropora tenuis* recruits that had settled on natural reef substrata inside the larval-enhanced plots became visible underwater 9 months after the larval enhancement activity. The concurrent size of the juvenile *A*. *tenuis* on the recruitment tiles and their characteristic morphology were used as the basis for identification of *A*. *tenuis* juveniles on natural substrata inside the plots that originated from the larval enhancement experiment. Identified *A*. *tenuis* juveniles on the natural reef substrata were mapped and tagged with a numbered tag for growth and survivorship monitoring together with the juveniles on the recruitment tiles for 36 months after the larval enhancement. Juvenile colonies on the natural reef substrata were repeatedly monitored at 9, 12, 15, 17, 19, 21, 23, 31 and 35 months after settlement.

*In situ* growth monitoring commenced at this time, with the length (*l*), width (*w*) and height (*h*) of each of the juvenile corals on recruitment tiles and natural substrata measured using calipers. Mean planar diameter was calculated from the maximum and minimum diameters measured for each colony. The ecological volume (EV) was calculated using the volume formula: EV = πr^2^ *h*, where r = (*l* + *w*)/4^45^.

### Background coral recruitment

Background natural recruitment rates in the restoration site were monitored by deploying and replacing ten 10 × 10 cm natural *Acropora* coral skeleton recruitment tiles quarterly in each of the control plots for 24 months from May 2013 to May 2015. Tiles were biologically conditioned for one month in tanks with flow-through seawater and aeration at the BML aquaculture facility then carefully transported in seawater to the experimental site. Tiles were labelled and attached to individual posts in the same manner as tiles used for the larval settlement experiment, described above. The recruitment monitoring periods encompass the known coral spawning periods in Bolinao-Anda reefs from February to July, with extended planulation periods throughout the year for brooding pocilloporid and poritid coral species^16,37^ (DWdC and PLH, pers. obs.). Retrieved tiles were bleached in 10% sodium hypochlorite solution for at least 48 hr and then air-dried. Tiles were examined under a stereomicroscope and each coral spat recorded was categorized as acroporid, pocilloporid, poritid, others, or unidentified (for broken and damaged unidentifiable recruits) based on their skeletal morphology^46^.

### Onset of sexual reproduction

The onset of sexual reproduction in *A*. *tenuis* colonies that recruited onto coral tiles and natural reef substratum was monitored by carefully breaking and replacing small branches to determine the presence of gametes^35^. Monitoring was done at age 10, 20 and 34 months prior to the predicted spawning periods after 1, 2 and 3 years of growth. After two years of growth, the *A*. *tenuis* colonies resulting from settled larvae ranged in size from 3 to 23 cm mean diameter, with an overall mean diameter of 11 (±1) cm, which was close to the minimum size threshold recorded for *Acropora* colonies to become sexually reproductive^32,33^. At 35 months the volume of all gravid and non-reproductive colonies was measured to determine the size and age of onset of sexual reproduction. At 36 months spawning of the gravid *A*. *tenuis* colonies was monitored *in situ* after the April 2016 full moon.

### Coral production cost analysis

To estimate the cost of producing sexual coral recruits from this study on Magsaysay reef we used the methods outlined in Edwards^16^. The costs were categorised and then summed for all materials and infrastructure, boat hire and fuel, diving and labour for gravid coral collection (using different wage rates for different skill levels), spawning and larval rearing, site preparation and capital costs for the larval mesh enclosures (Supplementary Table S1). To estimate the cost per coral colony produced at different ages, the total cost was divided by the total numbers of juvenile corals alive at 9 months and at three years of age in the four larval enhancement plots. Costs were in Philippine Pesos and were converted to US$ (Supplementary Table S1).

### Environmental parameters

Sea Surface Temperature (SST) data from 2013 to March 2016 were obtained from coralreefwatch@noaa.gov, and these data were supplemented with *in situ* data recorded on a Stowaway temperature data logger deployed at 3 m depth near the experimental plots from February to July 2015. Other water quality and environmental parameters were monitored periodically during the study (Supplementary Table S2). Salinity was measured with a handheld refractometer using three replicate water samples collected from the larval enhancement site in 15 ml tubes. Light intensity was measured using a LI-COR^®^193SA spherical quantum sensor attached to a LI-COR^®^ LI-1400 data logger. The average of ten to fifteen consecutive light readings at noon were obtained. Sedimentation was quantified using three PVC sediment traps, each 5 cm in diameter and 20 cm in height, conforming to the 1:4 ratio recommended by Gardner (1980)^47^. Sediment traps were deployed for 24 h periods in the larval enhancement site. Water motion was measured using the clod card technique^48^. Three clod cards were deployed for 24 h per plot, and in the BML seawater aquaculture tanks. Clod cards were weighed prior to deployment and after retrieval, and the weight lost was used as an indicator of the water motion.

### Statistical Analysis

Data are reported as mean values ± standard error. The four larval enhancement plots and the four control plots were used as statistical replicates (N = 4), with data from the ten tiles in each plot averaged to quantify mean initial settlement rates, and subsequent growth and number of surviving recruits at age 35 months. Analysis of similarities (ANOSIM) was performed using PRIMER v6 to test for similarities and significant differences in the percent benthic cover composition (e.g., sand, rubble, macroalgae, coral) between larval enhancement and control plots before the larval experiment. Benthic cover data did not require transformation and similarities were calculated using the Bray-Curtis similarity measure. Significant differences in the initial larval settlement patterns on different tile surfaces on tiles from the larval-enhanced plots after five days of larval settlement were tested using One-way ANOVA. Tukey’s HSD test was conducted post hoc to determine significant differences in settlement patterns among tile surfaces.

Survivorship of coral recruits on different surfaces of tiles was analysed using survival analysis, a non-parametric pairwise comparison test based on the Kaplan–Meier function^49^. The same analysis was used to determine any significant difference in survival patterns of juvenile corals on natural substrata and on tiles from nine to 35 months after the larval enhancement. In one case multiple recruits that had settled close to each other on the reef substratum subsequently grew together and fused to form a single colony, and this was considered as a ‘censored observation^49^. Significant increases in growth of juvenile corals through time were determined using Repeated Measures ANOVA. One-way ANOVA was used to compare growth rates of juvenile corals on recruitment tiles versus growth rates on natural substrata, and to compare the rates of natural recruitment of acroporids on settlement tiles in the study area versus recruitment of *A*. *tenuis* from the larval enhancement activity.

## Results

### Benthic cover and reef condition

**Figure 2:**
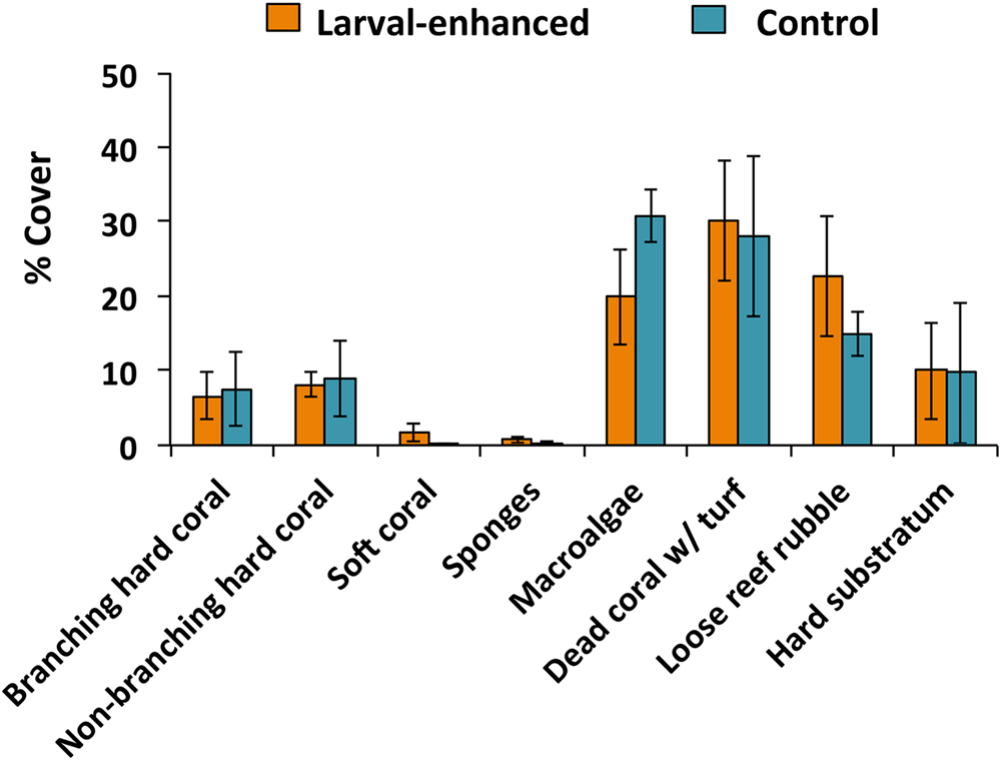
Mean percentage cover of benthic categories in larval-enhanced (N = 4) and control (N = 4) plots before the experiment. Error bars are ± SE.

Benthic cover and reef community status at the reef site were quantified prior to the experiment and were comparable (*R*: 0.04, *P* = 0.40, ANOSIM) in the four replicate larval enhancement plots and the four control plots (Fig. 2). All plots were degraded and characterised by low mean living scleractinian coral cover of 15.6% (±1.6% SE), with soft corals, sponges, macroalgae and dead coral covered with algae comprising 56.8% (±2.9%) mean benthic cover (Fig. 2). Dead coral and coral rubble surfaces potentially available for coral larval settlement equated to 27.6% (±3.7%) of mean cover, which represents about 7 m^2^ of the reef area within each of the 24 m^2^ plots (Fig. 2).

### Larval development and initial settlement

**Figure 3:**
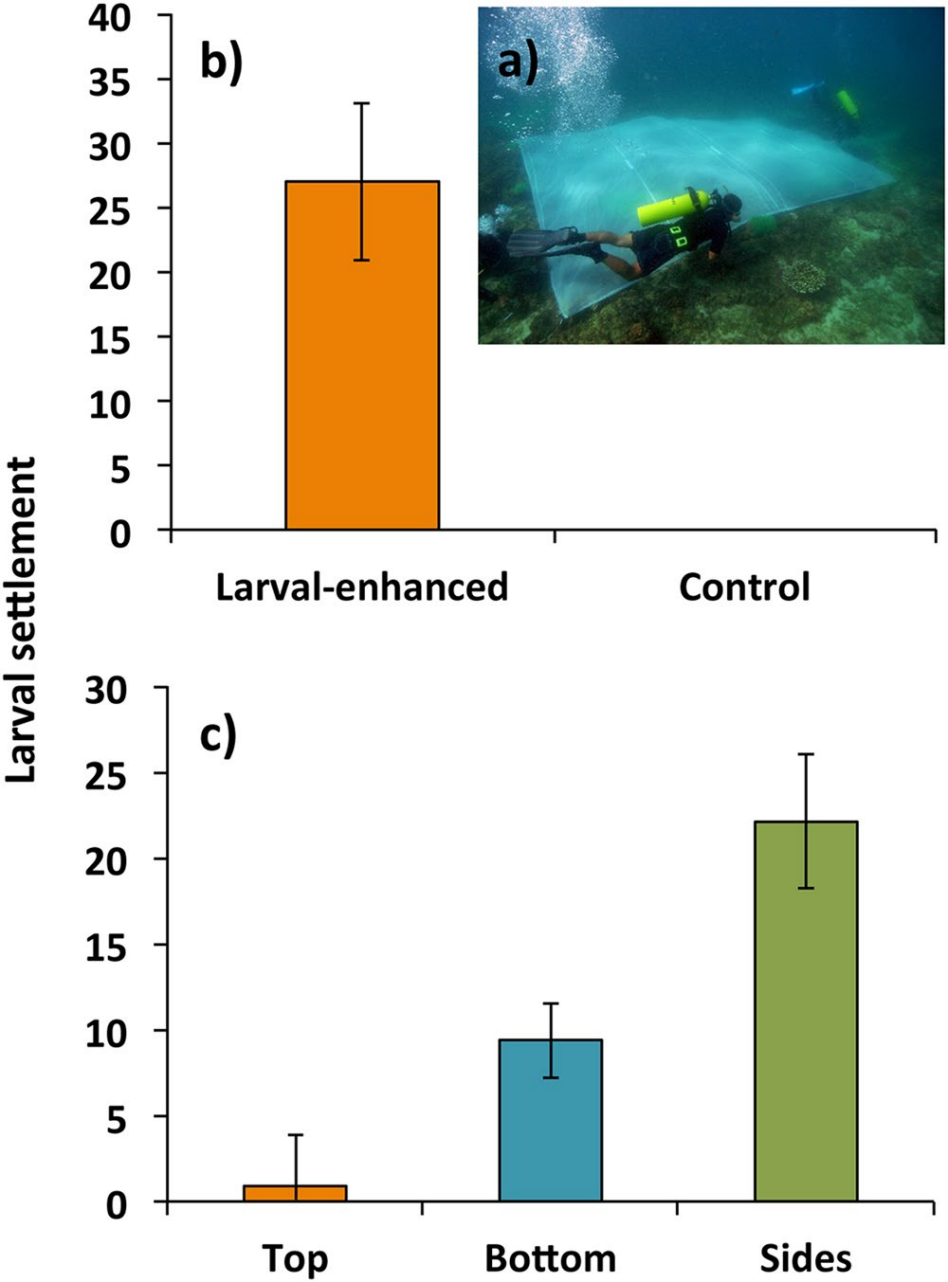
(**a**) Larval mesh enclosure used to retain larvae on reef plots during settlement, (**b**) mean initial *A*. *tenuis* larval settlement on all tile surfaces in the larval-enhanced (N = 4) and control (N = 4) plots after 5 days, and (**c**) mean settlement on the different surfaces of tiles in the larval-enhanced plots (N = 4). Means are for ten tiles per plot and averaged among the four replicate plots. Error bars are ± SE.

A total of 1,021 *A*. *tenuis* spat settled on the 40 10 × 10 cm (total of 14,400 cm^2^ tile area) biologically conditioned natural *Acropora* skeleton tiles that were attached to reef surfaces in the larval enhancement treatment plots (Fig. 3a) during the 5-day settlement period (Fig. 3b). Mean total settlement on the sets of ten tiles in each of the four larval enhancement treatment plots was 255.3 (±68.6) spat. This was significantly higher than for control plots in which no *A*. *tenuis* spat settled (Fig. 3b). Mean larval settlement on tiles in the larval enhancement plots was significantly higher on side surfaces of tiles compared to bottom and top surfaces (*α* = 0.05, Tukey’s test, Fig. 3c).

### Recruit survival

**Figure 4:**
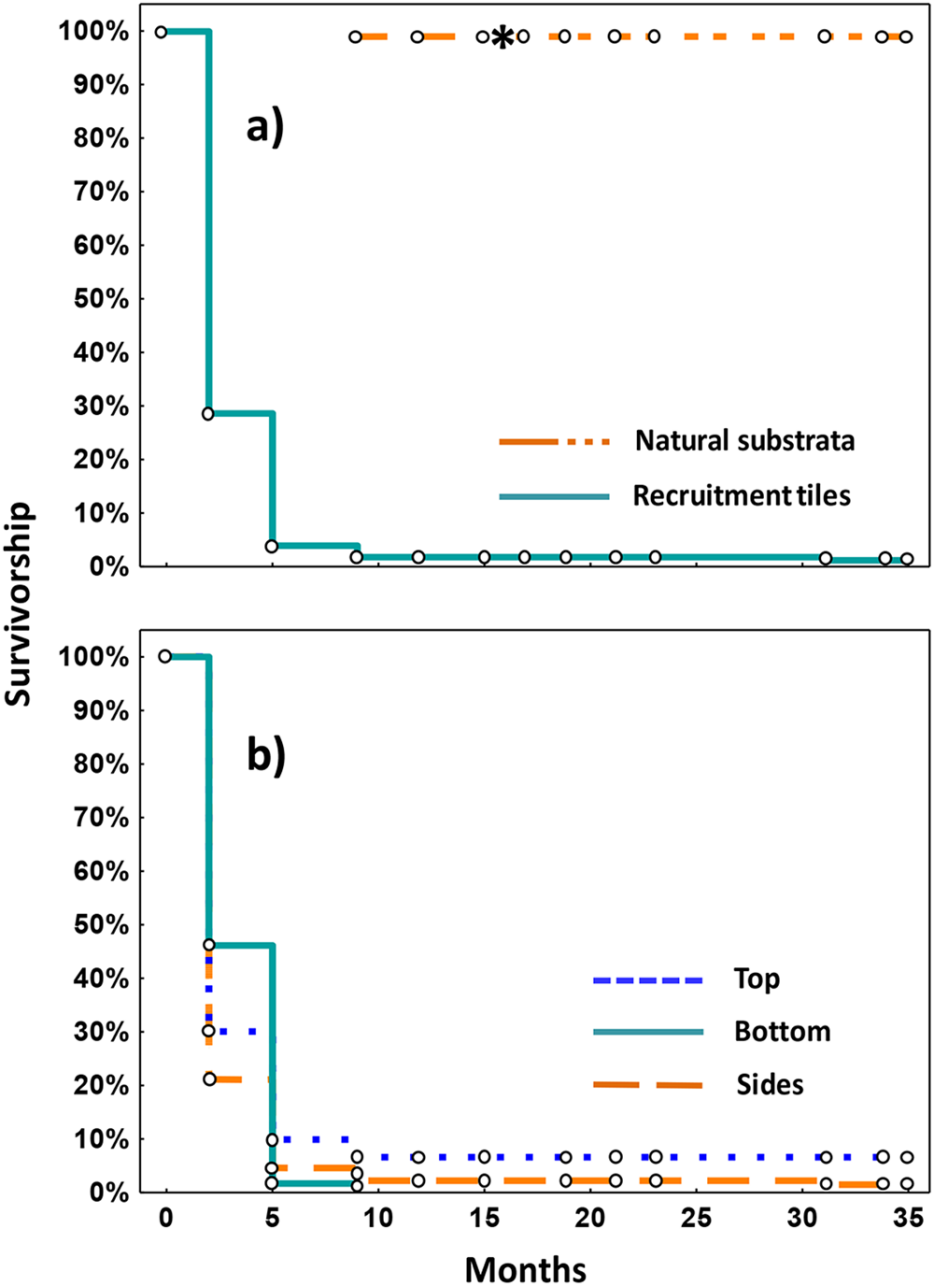
Kaplan-Meier survivorship over 35 months for (**a**) *A*. *tenuis* recruits settled on tiles (N = 1021), and for visible recruits on natural reef substrata starting at 9 months post-settlement (N = 94) where the *marks the monitoring period where 31 juvenile corals had fused to form a single colony, and (**b**) *A*. *tenuis* recruits on different tile surfaces (N = 1,021).

Subsequent repeated monitoring showed a steep decline in survivorship of settled juvenile corals on tiles during the first five months after settlement (Fig. 4a), corresponding to a Type III survivorship curve. Little additional mortality was recorded after five months and survivorship stabilised after nine months. This coincided with the period when the previously cryptic juveniles that had settled on the natural reef substrata became visible recruits at 2.1 (±0.4) cm mean diameter for *in situ* growth and survivorship monitoring (Fig. 4a). At nine months after settlement, a total of 94 *A*. *tenuis* recruits were recorded on the natural reef substrata in the four larval enhancement plots, and a total of 18 recruits survived on the settlement tiles.

For corals that settled on the natural reef substrata, all juvenile corals remained alive from nine months to 15 months post-settlement. There were 31 individual juvenile corals that had settled close together on the reef fused to form a single coral colony between 15 and 17 months of age (Fig. 4a), hence the fused corals were treated as a ‘censored’ observation in the survival analysis. Among coral recruits that settled on the natural reef substrata no mortality was recorded from 17 to 35 months after settlement, resulting in a total of 66 colonies surviving in the larval enhancement plots after almost three years.

**Figure 5:**
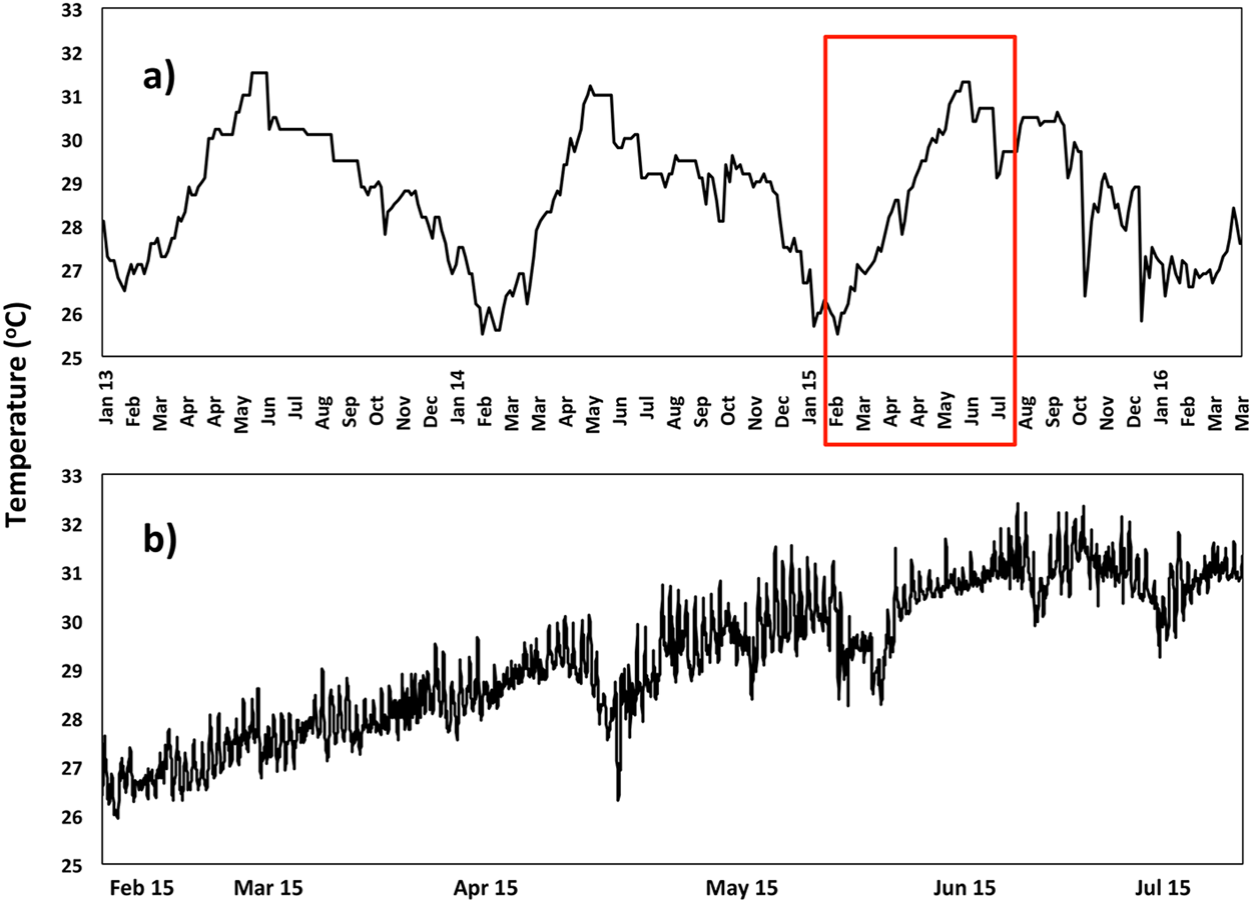
(**a**) Sea surface temperature profile in the Bolinao region during this study from January 2013 to March 2016 (coralreefwatch@noaa.gov), and (**b**) temperatures recorded *in situ* at the Magsaysay Reef study site using a data logger from Feb 2015 to Jul 2015, corresponding to the period indicated within the red box in (**a**).

There was 100% survival of tagged *A*. *tenuis* settled juveniles on recruitment tiles from nine months to two years post-settlement (Fig. 4a). However subsequent monitoring in 2015 and 2016 showed that five colonies on settlement tiles died between 24 and 32 months post-settlement (Fig. 4a,b). One colony appeared partly bleached during April 2015, coinciding with increasing sea temperatures on the reef (Fig. 5a,b), and this colony was dead when monitored in December 2015. Four larger colonies on tiles were eaten by *Drupella* sp. gastropods in December 2015, probably just prior to that monitoring period based on the low density of turf algae growing on the recently exposed coral skeletons and the presence of *Drupella* on these colonies. Another large colony on a tile was partially eaten by *Drupella* when monitored in December 2015, so the gastropods were removed and the remaining colony branches survived and grew. In contrast, no *A*. *tenuis* colonies on the natural reef substrata were eaten by *Drupella*. A total of 13 colonies survived on settlement tiles after 35 months.

**Figure 6:**
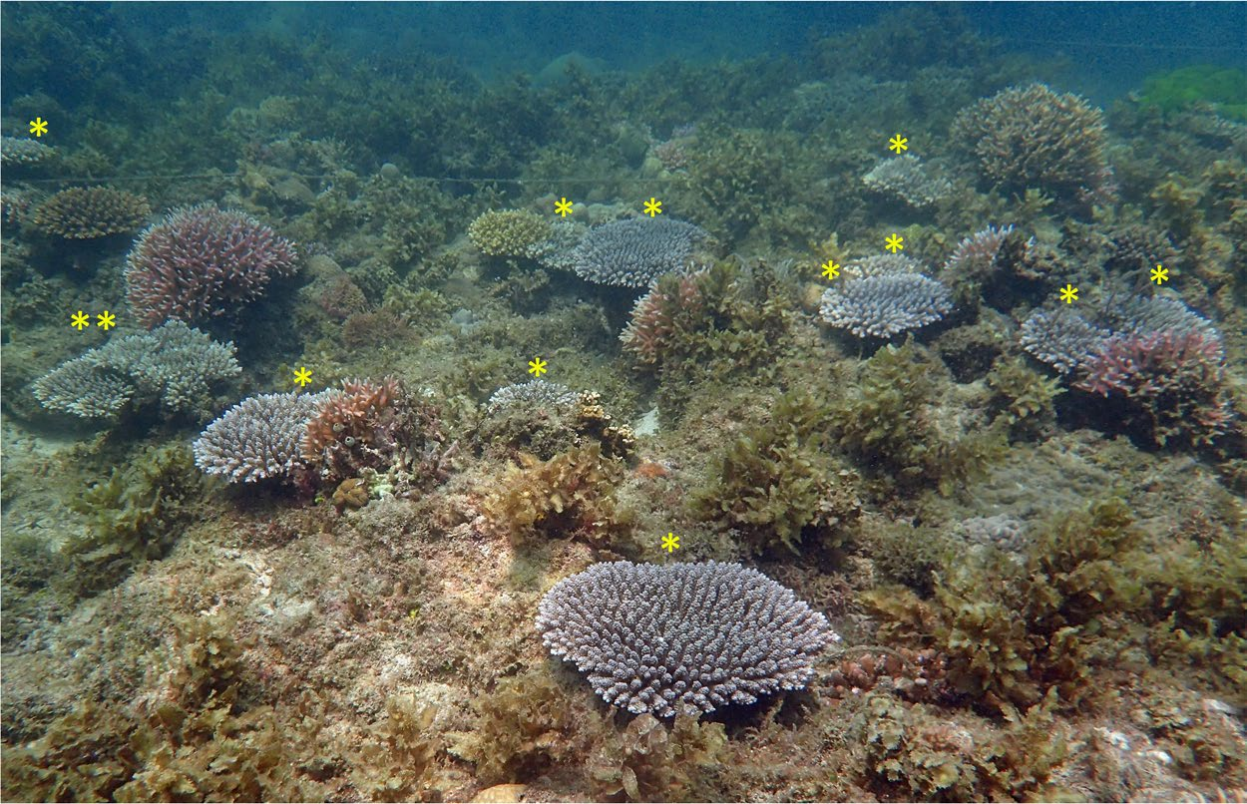
*Acropora tenuis* colonies growing on a larval enhancement plot three years after settlement. *Indicates colonies grown from settled larvae, **indicates the large colony derived from fusion of 31 small colonies grown from settled larvae.

The patterns of high survivorship of visible recruits on the natural reef substrata and on the settlement tiles after 5 months occurred despite an anomalous increase in sea surface temperature to 32 °C and partial bleaching of some corals on nearby reef areas at 26 months after settlement (Fig. 5a,b). Survivorship of recruits on tiles from May 2013 to April 2016 (Fig. 4b) varied significantly among tile surfaces (χ² = 53.35, *P* = <0.01, Log-rank test; sides >bottom = top). The mean total number of surviving recruits on the ten tiles in each settlement site after 35 months was 3.3 (±1.0), which equates to 9 colonies (±2.6) per m^2^ of tile surface. The mean number of surviving *A*. *tenuis* colonies on the natural reef substrata in each settlement site after 35 months was 16 (±5.7), which equates to 2.3 new colonies developing from larvae on each m^2^ of the approximately 7 m^2^ of available reef substrata in each of the larval settlement plots (Fig. 6).

### Recruit growth and onset of sexual reproduction

**Figure 7:**
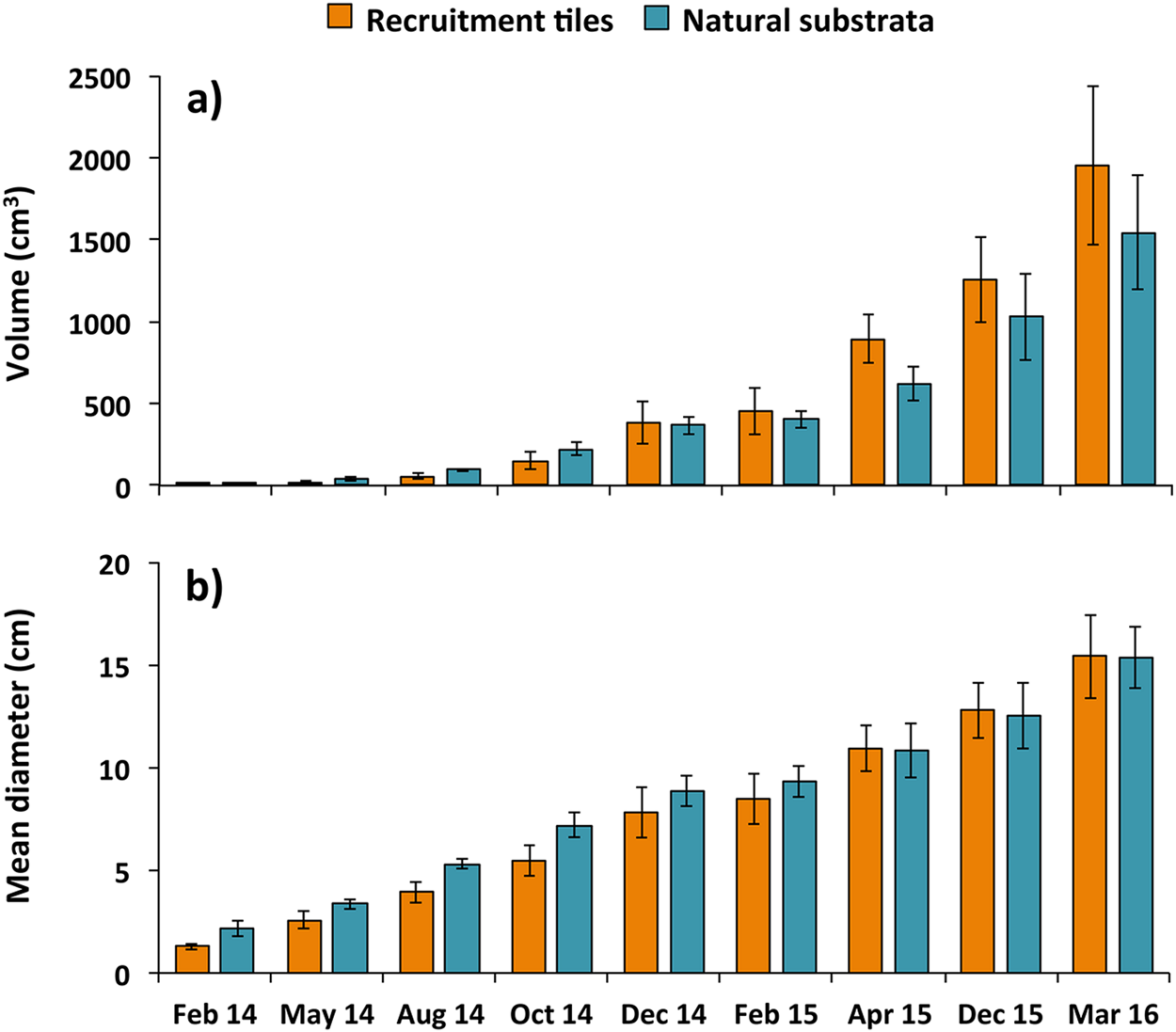
Mean volume (**a**) and mean diameter (**b**) of juvenile *A*. *tenuis* on recruitment tiles and natural substrata inside the four larval-enhanced plots (N = 4). Error bars are ± SE.

Average growth rates of *A*. *tenuis* recruits monitored on natural reef substrata from nine months to two years post-settlement reached 40.9 (±6.8) cm^3^ mo^−1^ and were similar (*F*_*3*,*6*_ = 2.36, *P* = 0.17, ANOVA) to growth rates of recruits that settled on tiles (59.3 ± 9.8 cm^3^ mo^−1^; Fig. 7). Volumes of recruits on tiles versus natural reef substrata did not differ significantly in any of the monitoring periods (repeated measures ANOVA; Fig. 7a). For growth analysis, data on growth of the 31 recruits that fused to form one colony (Fig. 6) were excluded. The volume for this fused colony reached 19232.5 cm^3^ with a mean diameter of 35 cm, which was the largest colony among the recruits (Fig. 7) and eight times larger than the mean volume of all the other 78 surviving corals after 35 months.

At nine months post-settlement, the 94 juvenile *A*. *tenuis* corals on natural reef substrata had a mean diameter of 2.1 ± 0.4 cm, and the 18 juvenile corals on tiles had a mean diameter of 1.3 ± 0.1 cm. From nine months to two years post-settlement, there was no mortality recorded for corals growing on natural reef substrata and on tiles, and mean diameters of two year old corals were 11.9 ± 1.0 cm and 10.9 ± 1.1 cm, respectively. After two years of growth, the *A*. *tenuis* colonies were not sexually reproductive. However, by 35 months of age colony size had increased up to 16.1 ± 0.7 cm mean diameter (Fig. 7b), and the 53 colonies that were larger than 12.5 cm mean diameter were sexually reproductive and spawned egg-sperm bundles after the April 2016 full moon. These comprised 44 reproductive colonies growing on the natural reef substrata and nine colonies on the tiles.

### Production costs

The total production cost for the sexually derived *A*. *tenuis* colonies in this study was US$1654.00. This equates to a production cost of $14.77 for each of the 112 recruits alive at 9 months, and $20.94 for each of the 79 colonies surviving after 35 months (Supplementary Table S1).

### Background coral recruitment

**Figure 8:**
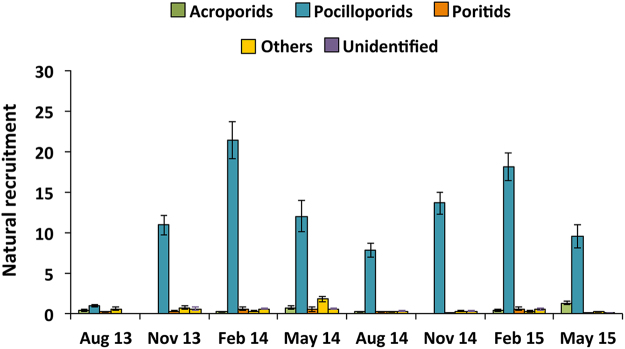
Mean natural coral recruitment patterns on monitoring plots (N = 4) in the larval enhancement study area, recorded on ten tiles combined per site. Error bars are ± SE.

A total of 4,197 coral spat were recorded settled on 307 natural recruitment tiles deployed for three-month periods over two years. Natural juvenile coral recruitment was dominated by pocilloporids (88.5%), with very minor recruitment of acroporids (2.7%), poritids (2.3%), and other recruits (3.8%), and 2.8% of recruits were broken and damaged and therefore unidentifiable (Fig. 8). Highest recruitment was consistently found during February monitoring periods which had means of 23 ± 2.5 recruits recorded on the ten tiles per plot in 2014, and 19.9 ± 1.9 recruits in 2015. Lowest recruitment rates were found during August 2013 (2.1 ± 0.3) and August 2014 (8.7 ± 1.0). Monitoring of adult corals and new visible colony recruits at the control plots over three years showed that no new natural *A*. *tenuis* coral recruits were present. *Acropora tenuis* recruitment from the larval settlement experiment was significantly higher (70 times greater) than natural acroporid recruitment during any of the monitoring periods (*F*_*8*,*27*_ = 19.20, *P* = <0.001, ANOVA; Tukey’s test, α < 0.05).

## Discussion

Despite increasing coral reef research and management activity, the global crisis in reef degradation and destruction continues to worsen and the loss of reef-building coral communities is occurring at unsustainable rates in many reef regions^4,7^. Natural recovery of coral populations and reef communities can occur where key threats are managed and disturbance regimes do not impair recovery^50^ and where natural rates of larval supply lead to sufficiently high rates of recruitment to enable the re-establishment of coral communities even after severe disturbances^51^. However, in many degraded reef areas current management is insufficient to control ongoing threats, and the loss of coral populations is so severe that larval supply is limited by the reduction in breeding corals and elevated rates of post-settlement juvenile coral mortality resulting from poor water quality and other chronic disturbances. Therefore, new active management approaches are needed for effectively slowing and reversing coral loss on degraded reef systems to promote recovery.

The loss of live coral cover and fish abundance on reefs in the Bolinao-Anda Reef Complex (BARC) in Northern Luzon since the early 1980s^27^ typifies the declining reef status in many regions in the Philippines and more broadly in southeast Asia over recent decades^2,7^. The BARC reefs were originally characterised by high live coral cover, fish abundance and high biodiversity^27^, however the experimental reef plots at Magsaysay reef and most nearby reef areas now have ‘very poor live coral cover’ *sensu* Wilkinson^2^. Furthermore, these reefs are now dominated by macroalgae, turf algae and dead coral (Fig. 2), consistent with a phase shift from coral to algal dominance^52^. Despite their poor status, environmental conditions at Magsaysay reef (Fig. 5, Supplementary Table S1) and other reefs nearby are sufficient for at least some adult corals to survive (Fig. 2) and the key threat of blast fishing has now been controlled^53^. However, the low rates of recruitment at the reef site during this study indicates that natural recovery of coral populations on these reefs will be slow and may not occur without active intervention^54^.

The results of this study demonstrate for the first time that mass larval supply resulting in enhanced rates of settlement directly onto badly degraded reef areas can significantly increase coral recruitment. As expected the highest rate of juvenile mortality occurred during the first few months after settlement, resulting in a Type III survivorship curve typical of many broadcast spawning marine invertebrates^55^ including corals^56–62^. The sources of early juvenile mortality could not be determined but are likely to include competition, overgrowth and potential allelopathic effects of algae^57,59^, competition with other reef invertebrates^63,64^, herbivore grazing and carnivore predation^65–67^, sedimentation and eutrophication^68,69^ and stochastic disturbances^50^. In addition, some coral recruits may have died after the first settlement census due to insufficient energy reserves remaining after settlement and initial skeletogenesis^58^, prior to the uptake and establishment of mutualistic *Symbiodinium* communities and the supply of photosynthetic energy-rich compounds^70^.

In contrast to some other field studies that were terminated due to low or no recruit survival, longer-term repeated monitoring during the present study showed remarkably high survivorship and growth of recruits occurred after five to nine months until 35 months (Fig. 4). This pattern of increased survival likely reflects the importance of early rapid growth to reach size-escape thresholds that result in increased survival and recruitment success^59,61,71,72^. High survival of recruits occurred after nine months with only five colonies that settled on tiles dying between 24 and 32 months post-settlement. Of these, only one of the *A*. *tenuis* colonies derived from the larval enhancement experiment bleached and died during the coral bleaching event at 26 months after settlement. Similar low rates of bleaching were observed among other *Acropora* colonies near the experimental site, with highest levels of bleaching in pocilloporid and fungiid corals. Predation by muricid *Drupella* corallivores resulted in the death of four of the larger colonies growing on tiles during December 2015, but no colonies growing on reef substrata were preyed upon. Predation by *Drupella* was also recorded to have reduced survival of some transplanted *A*. *millepora* juveniles on another BARC reef in a previous study^67^.

Mean growth rates of *A*. *tenuis* colonies resulting from settlement of reared larvae were similar on the natural reef substrata and coral skeleton tiles (Fig. 7) and these corals grew faster (5.2 cm yr^−1^) compared to cultured *A*. *tenuis* that were settled on artificial substrata in an outdoor hatchery, and subsequently transplanted after 18 months to reef habitats in subtropical Akajima Island, Okinawa, Japan (4 cm yr^−1^)^73^. Corals from the present study also grew faster than hatchery cultured *A*. *millepora* colonies where sub-adults were transplanted onto BARC reef outcrops (4.6 cm yr^−1^ mean diameter) or maintained in *in situ* nurseries for 3 years (4 cm yr^−1^ mean diameter)^67,74^. The relatively rapid growth rates of corals in the present study indicate the environmental conditions at Magsaysay reef are suitable for at least some corals, and that the surviving colonies are adapted to these conditions. Rapid growth may also have been enhanced by larvae selecting suitable natural microhabitat refuges and microbial communities directly on the reef, whereas larvae settled in *ex situ* or *in situ* nurseries will experience different physico-chemical and microbial conditions compared with those experienced by juvenile or sub-adult corals subsequently transplanted to different reef sites^67,73,74^. This may cause some transplanted colonies to be maladapted to their new environments and is an important issue that needs to be considered in future coral restoration projects involving transplantation of corals after settlement^75^.

The fastest growth and largest volume was recorded for the colony that resulted from the fusion of 31 individual juvenile corals on the reef between 15 and 17 months of age. This fused colony subsequently exhibited irregular growth morphology (Fig. 6) and patches of different coloured polyp tissues indicating that it is a chimera resulting from multiple genotypes. Intracolonial genetic variability and chimerism has been reported in some wild adult reef coral populations including other *Acropora* species^76,77^ and could be used to enhance coral restoration efforts. Fusion of different genotypes increases juvenile colony size and growth to larger size refuges thereby increasing survival potential unless competition among genotypes or tissue resorption and cell parasitism results in detrimental impacts^61^.

Rapid colony growth resulted in the establishment of a new breeding population of *A*. *tenuis* on the degraded reef site within three years after larval settlement (Fig. 6), and these colonies will increase the supply of gametes and larvae available for potential recruitment on other reefs in the Lingayen Gulf in the future. These recruits became sexually reproductive and spawned earlier and at smaller mean colony sizes than cultured and transplanted juveniles of *A*. *tenuis* from Akajima Island, where some surviving colonies became sexually reproductive at age 4–5 years when they had grown to 20–25 cm mean diameter^73^. The results from the present study support the earlier estimate of a minimum age at first reproduction of 3–5 years for *Acropora* recruits^78^, and are similar to the age and colony sizes at first reproduction for *A*. *millepora* recruits that were reared *ex situ* and transplanted onto BARC reef areas^74^.

Although low numbers of *Acropora* spat were recorded on recruitment tiles at the study site, no new visible *A*. *tenuis* natural recruits appeared in any plot during the three year study. This indicates that if any natural *A*. *tenuis* larvae were present and did settle on the reef substrata, none survived to visible size. These results indicate that natural background acroporid larval supply and recruitment are currently very limited at these degraded reef plots, hence these reef areas will have very slow rates of *Acropora* population recovery without active intervention. The lack of recovery at Magsaysay reef is also likely to be influenced by the availability of suitable substrata, potential exclusion or competition from other benthic organisms, and high post-settlement mortality of settled spat. Similar issues of natural recruitment limitation and failure have been documented on Caribbean reefs^79^ where coral cover and spawning biomass have been decreasing for some decades^13^. In these situations, reef recovery requires active intervention to increase the supply of genetically diverse larvae to ensure successful recruitment and survival at rates higher than the mortality of adult populations.

Transplantation of coral fragments on degraded BARC reefs has been shown to significantly increase the abundance and diversity of reef fish and macroinvertebrates^54,80^. Therefore, the re-establishment of complex three-dimensional habitats from the growth of reef-building corals from this study (Fig. 6) is likely to increase biodiversity and biomass of reef organisms on the restoration site, leading to improved reef status and ecosystem services^81^.

It has been noted that for future reef restoration interventions to have impacts at meaningful scales, they must be effective, relatively simple and affordable^16^. This study used organza cloth for the larval enclosures as this material is commonly available and relatively inexpensive, allows water to flow through the mesh to maintain larvae in a healthy condition and is sturdy enough to withstand intermittent strong surges on the reef site. The use of these low-cost enclosures helped to reduce the total production cost for this study to US$1654.00, and an average production cost of $20.94 for each of the colonies surviving after 35 months. The ~$21 cost for each three year old *A*. *tenuis* colony derived from larvae settled directly onto the reef plots and coral tiles is substantially less expensive than the production costs of 2.5 year *A*. *millepora* coral transplants that were held in an *in situ* nursery for 7 months (US$284), 14 months ($217) or 19 months ($61) prior to transplantation to reef sites^67^. Production costs for 2.5 year old *A*. *palmata* recruits ranged from US$325 for corals reared in a land-based nursery, to $13 for corals outplanted onto a reef after two weeks^82^.

Although post-settlement mortality is a key challenge and potential bottleneck for coral recruitment, our results clearly demonstrate that larval settlement and recruitment of *Acropora* coral species can be significantly enhanced using mass larval settlement to rapidly initiate coral population restoration and fast growth to sexual maturity within three years, even on degraded reef areas. Early post-settlement mortality can be ameliorated through the use of increased concentrations of competent coral larvae to substantially increase initial settlement rates and recruitment overall. This combined with high survival of juvenile corals indicates that mass larval enhancement is a viable and relatively low-cost active restoration option for initiating coral population recovery on degraded reefs, where environmental conditions are suitable for reef corals to grow but natural recruitment is limited, and chronic disturbances and key threats are effectively managed.

## Electronic supplementary material

Supplementary Information
