# Supplementary material for: Enhanced larval supply and recruitment can replenish reef corals on degraded reefs
Source: Sci Rep. 2017 Oct 25;7:13985. doi: 10.1038/s41598-017-14546-y (PMC5656657; doi:10.1038/s41598-017-14546-y)
Supplement: Supplementary file 1 — Supplementary Information [file 41598_2017_14546_MOESM1_ESM.pdf]

Supplementary Material

Enhanced larval supply and recruitment can replenish reef corals on degraded reefs

Dexter W. dela Cruz[a,b], Peter L. Harrison[a,*]

[*]Corresponding author.

email: peter.harrison@scu.edu.au

Table S1. Summary of costs for different activities related to larval enhancement and production of coral colonies. SCUBA gear hire, air tanks and boat rental from BML to the study site were based on 2013 rates. The BML outdoor hatchery facility and support system costs include equipment (seawater pump, air blower, pipe assemblies, sedimentation tank), maintenance and electricity. Costs were originally estimated in Philippine Pesos (PhP) and converted to US$ using the conversion rate: PhP45 = US$1. *total value divided over 10 years of use, **total value divided by two uses.

**Coral Production Activities**	**Days**	**Total (US$)**
I. Collection of gravid *A. tenuis* colonies	1	
A. Materials (including SCUBA equipment rental)		74.32
B. Boat rental		105.40
C. Hired labor (2 personnel)		39.11
Sub-total		218.83
II. Hatchery work	10	
A. Facility and support system		9.10
B. Culture tanks and accessories*		10.40
C. Hired labor (2 personnel)		357.80
Sub-total		377.30
III. Larval enclosures production**	~15	
A. Materials		145.40
B. Hired labor (3 personnel)		103.56
Sub-total		248.96
IV. Site selection and preparation	1	
A. Materials (including SCUBA equipment rental)		171.82
B. Boat rental		105.40
C. Hired labor (3 personnel)		54.67
Sub-total		331.89
V. Larval enhancement activity		
A. Materials (including SCUBA equipment rental)	1	253.94
B. Boat rental		105.40
C. Hired labor (10 personnel)		118.00
Sub-total		477.34
**TOTAL**		**1654.32**
**Cost per colony 9 months post-larval enhancement**		**14.77**
**Cost per colony 35 months post-larval enhancement**		**20.94**

Table S2. Environmental parameters measured at the Magsaysay reef study site during October 2013 to March 2015.

Environmental parameters	Oct 2013	Apr 2014	Jun 2014	Jul 2014	Sept 2014	Dec 2014	Mar 2015
Salinity (ppt)	32	34	34		30	34	34
Light (μE m^{-2} s^{-1})				584.4 ± 3.6	131.4 ± 2.3	857.5 ± 15	
Sedimentation (g $m^{-2}day^{-1}$)	0.2 ± 0.03	0.2 ± 0.01	0.2 ± 0.03		0.1 ± 0.01	1.4 ± 0.1	1.2 ± 0.1
Turbulence (g day^{-1} weight lost)	11.5 ± 0.7	10.9 ± 0.3	12.7 ± 0.2		19.2 ± 0.1	9.1 ± 0.4	12.7 ± 0.1
